# Supplementary material for: Fructosyl Amino Oxidase as a Therapeutic Enzyme in Age-Related Macular Degeneration
Source: Int J Mol Sci. 2024 Apr 27;25(9):4779. doi: 10.3390/ijms25094779 (PMC11083825; doi:10.3390/ijms25094779)
Supplement: Supplementary file 1 [file ijms-25-04779-s001.zip › ijms-2875744-supplementary.pdf]

Supplementary Figure S1

FAOD treatment on drusen surface (haematoxylin stained parafiin slice **of human retina, 10 x 40;** RPE = retinal pigment epithelium, BM = Bruch's membrane). Following FAOD treatment, the **mean surface** of the drusen (area marked by the blue contour) has been reduced to 45 ± 21 % of the initial surface (n=15, P < 0.005). All drusen showed a reduction in size upon FAOD treatment.

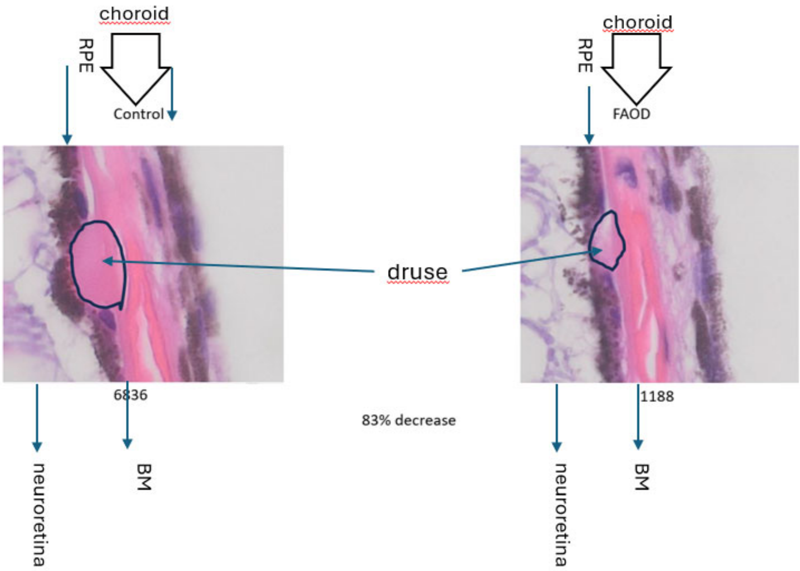

FAOD treatment on drusen surface (haematoxylin stained parafiin slice **of human retina, 10 x 40;** RPE = retinal pigment epithelium, BM = Bruch's membrane). Following FAOD treatment, the **mean surface** of the drusen (area marked by the blue contour) has been reduced to 45 ± 21 % of the initial surface (P < 0.005).

Drüse 2

Control

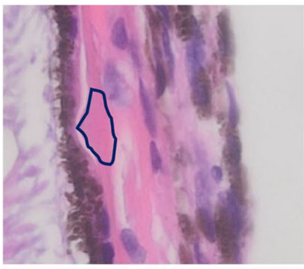

2734

FAOD

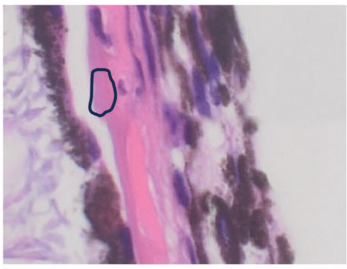

1047

62% decrease

Drüse 1

Control

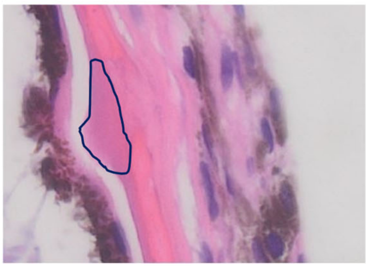

5470

FAOD

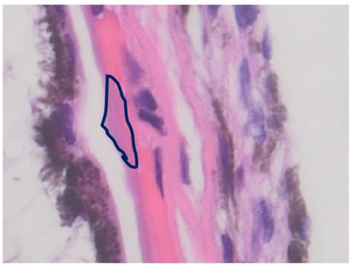

2094

62% decrease

Drüse 4

Control

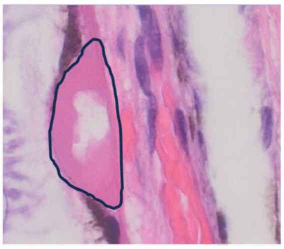

19361

FAOD

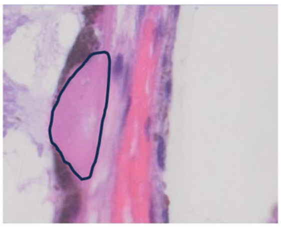

12709

35% decrease

Drüse 5

Control

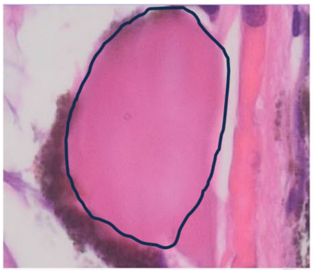

51326

FAOD

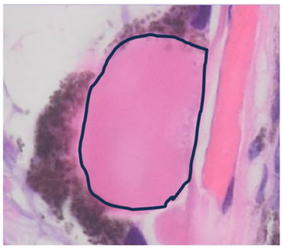

35689

31% decrease
